# Evaluation of the Korean version of the self-assessment of nursing informatics competencies scale

**DOI:** 10.1186/s12912-019-0392-5

**Published:** 2019-12-30

**Authors:** Kyoungsan Seo, Yul Ha Min, Seung-Hye Choi, Haeyoung Lee

**Affiliations:** 1 Nursing Department, College of Health Sciences, Cheongju University, 298 Daeseong-ro, Cheongwon-gu, Cheongju-si, Chungcheongbuk-do Republic of Korea; 2 College of Nursing, Gachon University, 191 Hambakmoero, Yeonsu-gu, Incheon, Republic of Korea; 3 Red Cross College of Nursing, Chung-Ang University, 84 Heukseok-ro, Dongjak-gu, Seoul, 06974 Republic of Korea

**Keywords:** Informatics, Validation studies, Nursing students

## Abstract

**Background:**

In order to assess nursing students’ informatics competency, we need a comprehensive Korean version scale that reflects the important advances in nursing informatics and can make up for the lack of an existing measure. This study aimed to cross-culturally adapt the Self-Assessment of Nursing Informatics Competencies Scale (SANICS) into Korean (K-SANICS) and verify its validity and reliability with nursing students.

**Methods:**

The design of this study was a methodological approach to translate and evaluate the Korean version tool (K-SANICS). A total of 254 nursing students at four universities in Korea completed a structured questionnaire including background characteristics and the K-SANICS. Reliability and validity of the 30-item K-SANICS were evaluated using Cronbach’s α, content validity, factor analysis, and contrasted groups approach.

**Results:**

Cronbach’s α was .95. Exploratory factor analysis was performed to verify the scale’s construct validity, identifying 30 items across six categories: advanced skills for clinical informatics, basic application skills, basic computer skills, roles in nursing informatics, skills for clinical applications, and attitude toward computers in nursing.

**Conclusion:**

The K-SANICS may be used as a reliable assessment tool of nursing students’ nursing informatics competencies. It is expected that the K-SANICS will contribute to establishing, operating, and evaluating nursing informatics curricula and also can be used in a clinical setting.

## Background

Remarkable advances in information technology have led to the implementation of systems utilizing a diversity of such technologies in healthcare settings. For example, the Electronic Nursing Record system, which was first implemented in Korea in 2003, had reached a penetration rate of 70% as of 2012 [1]. Other systems, like the Electronic Medical Record and Electronic Health Record systems, facilitate nurses in carrying out patient-centered care by providing health information recorded by multiple health professionals and patients themselves. Nurses can also promote patient safety and quality care by using clinical decision-making support systems and technology-based tools such as barcodes and mobile devices [2].

With this trend in the current healthcare environment, informatics competency, which refers to the ability to perform nursing tasks and roles related to informatics activities, is one of the fundamental competencies demanded of nurses [3, 4]. This requires improvement though new curricula and continuous training in clinical settings. Various curriculum contents have been tried, but these were not provided in all universities nationally. In 2014, the rate of nursing informatics education was just 64.7% in undergraduate curricula and 28.1% in graduate curricula at nursing schools in Korea [5]. Furthermore, whereas various information technologies, such as computers, smartphone applications, and artificial intelligence, are increasingly applied in clinical settings, their implementation in nursing education in Korea has been piecemeal, e.g., the Electronic Nursing Record system offered as a mobile application for students [6] ethical decision-making using the computer [7], and standard terminology system using a smartphone application [8]. In addition, the rapid development rate of information technology may have opened a gap between educators and nursing students in their basic knowledge and use of informatics. This may be an obstacle for educators to accurately grasp the needs of students in relation to nursing informatics. Therefore, a broad understanding of nursing informatics competency is essential prior to developing nursing curricula.

There are several instruments to measure nursing informatics competency, and the concepts they encompass vary widely. The Nursing Informatics Competency Questionnaire by Staggers et al. [4], which has been widely used in studies in Korea and abroad [9–14], measures nurses’ competencies in terms of informatics knowledge and skills. On the other hand, the Competencies in Pre-licensure Nursing Education, developed by the Quality and Safety Education for Nurses (QSEN) project in the United States, include informatics knowledge, skills, and attitudes but not computer knowledge or basic computer skills [13]. Recently, informatics competency has been defined as a comprehensive concept that encompasses knowledge of and ability to use and integrate computer science, information science, and nursing science, which led to arguments that scales for measuring nursing informatics competency must also cover computer skills and informatics knowledge and abilities [15].

Nevertheless, existing informatics competency scales did not reflect important advances in the field, such as standardized terminology, which is key for sharing and reusing data from the Electronic Medical Record and Electronic Health Record systems, and wireless communication, which is essential for using mobile health devices and wearable health trackers. Thus, these factors were included in the development of the Self-Assessment of Nursing Informatics Competencies Scale (SANICS) in 2009 [16]. The SANICS includes items for the role of clinical informatics in nursing (Factor 1; items 1–5), basic computer knowledge and skills (Factor 2; 6–20), applied computer skills (Factor 3; 21–24), clinical informatics attitudes (Factor 4; 25–28), and wireless device skills (Factor 5; 29–30) [16].

The SANICS was validated and was internally consistent for nursing students with various educational and demographic backgrounds. Moreover, the high responsiveness of the SANICS indicates its strong ability to detect significant changes in informatics competencies [17]. However, as the SANICS was developed in the United States, its direct use with Korean students is not appropriate. When applying instruments developed in different languages and cultural environments, it is important not only to accurately translate the items of the scale linguistically but also to ensure cultural appropriateness to maintain content validity at the construct level. Items that are culturally and situationally inappropriate may be revised; however, doing so may alter the psychometric properties of the instrument. In such cases, the adapted instrument may require additional analyses to establish reliability and validity [18]. Therefore, this study aimed to cross-culturally adapt the SANICS into Korean and verify its reliability and validity to assess nursing informatics competency in the nursing field and future studies assessing nursing informatics competency in Korea.

## Methods

### Study design

The design of this study was a methodological approach to culturally adapt and evaluate the Korean version SANICS with a multi-regional cross-sectional survey.

### Setting and sample

Study participants were third- and fourth-year nursing students attending nursing schools offering a 4-year nursing bachelor program. Four universities of three provinces were selected with convenient sampling procedure. We only included third- and fourth-year students, who had exposure to hospital information systems during their clinical training. First and second-year students who did not participate in clinical practice were excluded.

There are diverse opinions and empirical rules about the appropriate sample size for factor analysis [19]. A sample size greater than 200 participants or a case-to-variable ratio greater than 5:1 are within a safe range [20]. Considering the appropriate sample size and dropout rate, we aimed to collect data on 330 students, and questionnaires were finally distributed to 300 individuals. Students who did not provide informed consent to participate in the study or answered incompletely were excluded.

### Ethical considerations

This study was approved by the Institutional Review Board of Hoseo University (no. 1041231–151,229-HR-036-07). Participants were recruited through advertisements on school boards. Students who wished to participate in the study gathered at the study briefing. The assistants provided a hard copy with information about the purpose and aim of the study, confidentiality and anonymity of participation, voluntary refusal to participate or study withdrawal, and harm and benefits, even after providing consent and completing the survey. An information and consent form approved by the IRB was used. To eliminate any coercion that might occur in the professor-student relationship, one of the researchers, not the professor of the school, explained the purpose of the study in detail. The survey was conducted only for those who understood this information and agreed to participate and completed a written consent form. The informed consent forms and completed questionnaires were stored in an electronic format in the researcher’s locked computer in the laboratory. Personal information that may identify study participants (except for gender and age) was neither collected nor used in the results reporting. All study data, including consent forms was managed in accordance with the Bioethics and Safety Act [21]. The above information was included in the explanation provided to the study candidates.

### Measurements: development of K-SANICS

The development process of the K-SANICS involved translation, reverse translation, revision, content validity verification, and a pilot survey with preliminary items.

#### Translation process

In this study, a four-step conceptual review process was applied based on the guidelines proposed in previous studies [18, 22]. Four steps were carried out in the order of translation, back-translation, expert committee review, and pre-test. After obtaining the author’s permission to use the original SANICS, a member of this study team, reviewed the original 30 items and translated them into Korean, and a bilingual expert translated them into English again without using the original version in English. The back-translated tool was approved by the original developer by email after several word revisions. The revised words were reflected in the Korean version.

#### Content validity

In order to establish the content validity of the preliminary measure, a panel of four experts, including specialists with expertise in nursing education and clinical nursing informatics currently working at a hospital in Korea, reviewed the preliminary items and rated each of them using the content validity index (CVI). They evaluated the relevance and suitability of all 30 items of the K-SANICS using a 4-point Likert-type scale (0 = *not relevant* and 1 = *very relevant*).

#### Preliminary K-SANICS

The preliminary K-SANICS includes 30 items and a 5-point Likert-type scale (1 = *cannot do it at all to* 5 = *can do it very well*) in Korean. There were no reverse score items. The higher the score, the higher the competency.

#### Pilot survey

The preliminary K-SANICS was pilot-tested with a sample of 15 nursing students from one of the study universities, who answered the questionnaire and were asked about the clarity of the translated items, if it was easy to understand, and the appropriateness of the tool form. The participants required 5–10 min to complete the measure and did not report any difficulty in understanding or responding to the items. The participants in the pilot survey did not participate in the study.

### Data collection

Data were collected from the target population, nursing students, from March 15 to April 30, 2016, using the structured questionnaire. The questionnaire includes K-SANICS items, gender, age, satisfaction with major in college, education experience with informatics, and need level for informatics training. One of the researchers, not the professor of the school, explained the purpose of the study in detail. The survey assistant provided a hard copy of information and the questionnaire. The informed consent forms and completed questionnaires were collected in a box prepared in advance. Students participated in the survey only once, and it took 15–20 min to complete the questionnaire. A small gift was given to the participants after they submitted the completed questionnaire.

### Data analysis

Data were analyzed using SPSS 20.0 version and AMOS 19.0, with a significance level of .05 (two-sided).

#### Participant characteristics

Background characteristics were analyzed using descriptive statistics. The mean and standard deviation of each item of the K-SANICS were obtained, and the total scores were calculated.

#### Reliability

Reliability of the K-SANICS was evaluated by using Cronbach’s alpha for internal consistency and corrected item-total correlations for the item analysis [23].

#### Validity

With respect to validity of the K-SANICS, three types of validity tests were performed: content validity was analyzed by experts, exploratory factor analysis with rotated varimax and the contrasted group approach was used to assess construct validity [23]. We calculated item-level CVIs, which refer to the proportion of content experts giving an item a relevance rating with 4 point-likert scale, and a scale-level CVI, which was calculated as the average item-level CVI for all items on the scale [24]. The Kaiser-Meyer-Olkin (KMO) measure for sampling adequacy and Bartlett’s test of sphericity were conducted, and factors with an eigenvalue > 1.00 were extracted in the exploratory factor analysis. KMO measures the degree of multicollinearity and should be greater than .50–.60, and Bartlett’s test is a measure of the probability that the initial correlation matrix is an identity matrix and is normally < 0.05. To confirm the differences between groups with contrasting experiences related to computers, t-tests were performed.

## Results

### Background characteristics of participants

Of the 254 participants, 89.0% were women, and the average age was 22.25 years (range 21–24). The mean score for satisfaction with major was 3.91 out of 5. A total of 48.0% of the students had related informatics education experiences at school or at the academy school, and 32.7% had computer-related certifications. The need for computer education and informatics education by nursing students was high, with 82.3 and 87.8%, respectively (Table 1).

**Table 1:** Background Characteristics of the Participants (*N* = 254)

Characteristics	Categories Or range	n (%) or Mean ± SD
Gender	Female	226 (88.98)
	Male	28 (11.02)
Age (years)	20~36	22.25 ± 1.80
Satisfaction with major in college	1~5	3.91 ± 0.81
Education experience with informatics	Yes	122 (48.03)
	No	132 (51.97)
Computer related Certification	Yes	82 (32.28)
	No	172 (67.72)
Need for computer education	Yes	209 (82.28)
	No	45 (17.72)
Need for informatics education	Yes	223 (87.80)
	No	31 (12.20)

*SD* Standard deviation

### Level of nursing informatics competences of participants

Of the 30 K-SANICS items, those with the highest scores were items 7 (searching and downloading contents of interest) and 30 (using wireless devices for data input) with an average score of 4.35 and 4.33 on a 5-point scale. The items with the lowest scores were items 1 (participating in the processes of selection, design, application, and evaluation of an information system in future clinical services) and 2 (encouraging myself and others to use information systems or application programs), with an average score of 2.99 and 2.85, respectively (Table 2).

**Table 2:** Item Analysis and Reliability of the Korean Version of the Self-Assessment of Nursing Informatics Competencies Scale (*N* = 254)

Item		Mean	SD	Corrected item-total correlation	CVI
1	(As a pre-service nurse), I am able to participate in the processes of selection, design, application, and evaluation of an information system in future clinical services.	3.00	.98	.51	1.00
2	(As a pre-service nurse), I am able to encourage myself and others to use information systems or application programs.	2.85	.95	.54	1.00
3	(As a pre-service nurse), I am able to improve the integrity and accessibility of information with considering confidentiality and legal, ethical and security issues but not restricted by them in future clinical services.	4.01	.91	.57	1.00
4	(As a pre-service nurse), I am able to find resources in the computer available for ethical decision making in future clinical services.	3.63	.85	.58	0.88
5	(As pre-service nurse), I am able to play the role of a supporter for the leader (manager) so that changes and the concept of information science may be incorporated into my professional area in future clinical services.	3.33	.94	.51	1.00
6	I am able to use electronic telecommunication devices (e.g. modem and other devices) for communication with other systems (e.g. accessing, uploading, or downloading information).	4.08	.87	.71	1.00
7	I am able to search and download contents of interest.	4.35	.78	.68	1.00
8	I am able to use a database program to build a simple database or to make a table.	3.97	.91	.69	1.00
9	I am able to use a database application program to enter and search information.	3.70	1.02	.72	1.00
10	I am able to do online literature search.	4.29	.80	.74	1.00
11	I am able to use a presentation program (e.g. PowerPoint) for making slides or screens.	4.15	.84	.73	1.00
12	I am able to use a multimedia presentation program.	3.79	.98	.65	1.00
13	I am able to use a word processor.	3.83	.98	.69	1.00
14	I am able to use a communication network (e.g. file server, www) for system search.	4.12	.86	.73	1.00
15	I am able to use an operating system. (e.g. folder copy, deletion, edition)	3.65	1.07	.58	1.00
16	I am able to use computer peripheral devices (e.g. CD-ROM).	3.73	1.06	.70	1.00
17	I am able to use a computer system in a safe way.	3.88	.96	.76	1.00
18	I am able to manipulate the Windows system (e.g. file handling using the File Manager, printer setting, access to an installed program, folder creation and deletion).	3.64	1.14	.65	1.00
19	I am able to view the basic configuration of a computer system (e.g. the specifications of a PC, high-performance computer, or professional computer).	3.05	1.20	.49	1.00
20	I am able to solve minor problems in application programs.	3.21	1.07	.63	1.00
21	I am able to use an application program for diagnosis coding.	3.24	1.10	.56	0.94
22	I am able to use an application program for developing a test tool.	3.41	1.03	.65	1.00
23	I am able to log in the patient medical information system (authorization using student ID and PW).	4.19	.89	.69	0.94
24	I am able to search data in the patient medical information system (authorization using student ID and PW).	4.15	.89	.66	1.00
25	I believe that health computing, namely, computer-based medical services will be popularized further.	4.13	.88	.63	1.00
26	I believe that the computer is merely a tool for providing better nursing services, and it cannot replace human functions.	3.64	1.09	.37	1.00
27	I do not think that a nurse has to be a computer programmer for the effective use of the computer.	3.31	1.05	.35	1.00
28	I believe that it is worthwhile for a nurse to participate in the design, selection, application, and evaluation of application programs and information systems used for health care services.	3.84	.90	.53	1.00
29	I am able to use wireless devices (e.g. PDA, smart phone) in order to search and download resources for patients’ safety and quality nursing services.	4.20	.85	.68	1.00
30	I am able to use wireless devices (e.g. PDA, smart phone) for data input.	4.33	.81	.72	1.00
Total					0.99/ave

*SD* Standard Deviation, *CVI* Content Validity Index, *CD-ROM* Compact Disc Read Only Memory, *ID* Identification, *PW* Password, *PDA* Personal Digital Assistants

### Reliability

Cronbach’s alpha was .95 (Table 2). Corrected item-total correlation coefficients for each item ranged from .35 to .76 (Table 2).

### Validity

#### Content validity

It resulted that the CVI of one item was .68, and two experts thought that this item was difficult to understand correctly. So, the word of the item was slightly modified by the researcher. There were no deleted items. A subsequent content validity test was performed by the panel. The index of all items ranged between .88 and 1.00 on average (Table 2).

#### Exploratory factor analysis

Exploratory factor analysis with rotated varimax was performed to assess the construct validity of the K-SANICS. The KMO measure for sampling adequacy was high (.93), and Bartlett’s test of sphericity was significant (χ^2^ = 5847.58, *p* < .001). Thus, the study data were appropriate for the factor analysis. Six factors of the K-SANICS with eigenvalues > 1.00 were extracted: We named Factor 1 “advanced skills for clinical informatics” (items 6, 7, 10, 23, 24, 25, 28, 29, 30), as the items included in the factor measure higher-level skills used in clinical settings, beyond basic computer skills. We named Factor 2 “basic application skills” (items 8, 9, 11, 12, 13, 14), as the items measure the skills to apply basic information technology. We named Factor 3 “basic computer skills” (items 15, 16, 17, 18, 19, 20), as it includes items that measure such skills. Factor 4’s name was “roles in nursing informatics” (items 1, 2, 3, 4, 5), as it includes items that measure roles as a pre-service nurse in clinical systems. We named Factor 5 “skills for using clinical applications” (items 21, 22), as it includes items that measure practical application skills in clinical settings. Finally, Factor 6 was named “attitude toward using computers in nursing” (items 26, 27), as it includes items that measure the students’ attitude toward using computers in nursing and informatics competence (Table 3).

**Table 3:** Exploratory Factor Analysis with Rotated Varimax of the K-SANICS (N = 254)

Factors	Item	Factor loading	Eigen value	Variance (%)	Cumulative variance (%)	Cronbach’s alpha
Factor1 Advanced skills for clinical informatics			13.17	43.91	43.91	.93
	24	.80				
	29	.79				
	30	.79				
	23	.78				
	25	.70				
	7	.69				
	10	.61				
	6	.57				
	28	.56				
Factor 2 Basic application skills			2.52	8.40	52.31	.92
	12	.79				
	13	.74				
	11	.74				
	8	.71				
	9	.66				
	14	.56				
Factor 3 Basic computer skills			1.96	6.53	58.84	.89
	19	.82				
	18	.78				
	20	.75				
	17	.67				
	16	.65				
	15	.58				
Factor 4 Roles in nursing informatics			1.42	4.75	63.59	.84
	2	.78				
	1	.78				
	5	.74				
	4	.68				
	3	.55				
Factor 5 Skills for using clinical applications			1.15	3.85	67.44	.86
	21	.74				
	22	.69				
Factor 6 Attitude about using computer in nursing			1.09	3.62	71.05	.64
	27	.81				
	26	.80				

*K-SANICS* Korean version of the Self-Assessment of Nursing Informatics Competencies Scale

The principal component analysis was followed by parallel analysis and item reduction techniques, resulting in a six-factor model that explained 71.0% of the total variance. Each factor accounted for the total variance as follows: 43.9% (Factor 1), 8.4% (Factor 2), 6.5% (Factor 3), 4.7% (Factor 4), 3.8% (Factor 5), and 3.6% (Factor 6). The factor loadings of the items ranged from .55 to .82, indicating correlations among the items. Cronbach’s alpha for the extracted factors ranged from .64 to .93 (Table 3).

#### Contrasted group approach

Differences in students’ nursing informatics competence according to experience characteristics were examined. The K-SANICS scores of students with informatics education experience were higher than that of the inexperienced students (t = 4.60, *p* < .001) (Table 4). There was a difference in the total K-SANICS score depending on computer certification (t = 4.99, *p* < .001) and the level of satisfaction with major (t = 4.76, *p* < .001) (Table 4).

**Table 4:** Difference of the K-SANICS Scores Between Contrasted Groups (*N* = 254)

Characteristics of group	K-SANICS total (Mean ± SD)	*t*	*p*
Education experience with informatics			
Yes (*n* = 122)	118.07 ± 16.55	4.60	<.001
No (*n* = 132)	107.73 ± 17.37		
Certification on computer			
Yes (*n* = 82)	120.68 ± 17.22	4.99	<.001
No (*n* = 172)	108.82 ± 18.04		
Satisfaction with major in college			
Yes (≥4) (*n* = 187)	115.88 ± 17.13	4.76	<.001
No (< 4) (*n* = 67)	103.79 ± 19.72		

*SD* Standard Deviation, *K-SANICS* Korean version of the Self-Assessment of Nursing Informatics Competencies Scale

## Discussion

This study translated the SANICS into Korean, modified the scale for use with Korean nursing students, and verified the validity and reliability of the adapted scale for measuring nursing informatics competency in nursing students.

The original SANICS has five domains—clinical informatics role, basic computer knowledge and skills, applied computer skills: clinical informatics, nursing informatics attitudes, and wireless device skills—with 30 items [16]. On the other hand, the K-SANICS has six factors—advanced skills for clinical informatics, basic application skills, basic computer skills, roles in nursing informatics, skills for using clinical applications, attitude about using computers in nursing—with 30 items. In particular, the factor 4 domain, “role in nursing informatics,” the difference being that the K-SANICS is for nursing students as opposed to clinical nurses.

Utley-Smith (2004) mentioned that nursing informatics competency is one of the five essential competencies that every nurse should possess [25]. Staggers et al. classified nursing informatics competencies into three domains: computer skills, informatics knowledge, and informatics skills [4]. In the present study, Factors 1 and 2 correspond to informatics skills, Factor 3 to computer skills, and Factor 4 to informatics knowledge. In addition to these three basic domains, our scale includes the skills for using clinical applications (Factor 5) and attitude toward informatics use and competence (Factor 6). Considering the rapidly evolving healthcare information technology environment, it is desirable to include items about attitudes toward informatics and application skills in addition to computer and informatics skills.

Changes in the factor composition of the K-SANICS in relation to the original instrument were not anticipated. Unlike the original instrument, the K-SANICS reflects latest information technologies, such as wireless communication [9–14]. However, our analyses revealed that wireless device skills are not an independent factor but are included in other factors. This suggests that wireless technology is highly universalized in Korea, and Korean undergraduates, who are mainly in their early 20s, do not perceive this technology as different from other information technologies. In fact, the annual education statistics reports of the Organization for Economic Co-operation and Development showed that Korean students are ranked number one in computer-based problem-solving [26], and mobile social media usage exceeds 60% among both male and female Koreans in their 20s [27].

The original SANICS comprises perception about one’s role as a nurse, basic computer knowledge and skills, application and wireless skills, and attitudes. Moreover, the items in SANICS reflect interprofessional informatics skills that support interpersonal communication, patient-centered care and team collaboration [28]. However, our analysis identified advanced skills, basic application skills, basic computer skills, clinical application skills, role, and attitude, which shows that skills were classified more specifically in the K-SANICS. In this study, we adapted an instrument developed for U.S. nurses to be used with Korean nursing students, and in the process, we revised some items to make them appropriate for our target population. Therefore, the changes in scale composition may be attributable to the adaptation process; however, we believe that the six factors that were newly extracted represent more specifically the computer and informatics skills that comprise nursing informatics competency. Further research is required to compare the results of this study by applying it to a non-Korean culture.

In the K-SANICS, the reliability of the 30 items was also high, with a Cronbach’s alpha of .95. Corrected item-total correlation coefficients for each item ranged from .35 to .76. As a reliability coefficient greater than .60 is sufficient in exploratory studies [29]. We can conclude that the scale measured the targeted concepts accurately and consistently.

In the contrasting group analysis, students who had received informatics education had higher K-SANICS scores than did those without previous education. Furthermore, students with computer certification and those with higher satisfaction with their major showed higher K-SANICS scores. In a previous study that compared graduate and undergraduate students using the SANICS, both groups showed similar scores in basic computer skills, attitudes about informatics, and wireless communication skills, but graduate students scored slightly higher on clinical informatics [30]. Because informatics competency is influenced by various factors, nursing educators should provide customized education that considers these factors, including students’ computer skills. In addition, it is necessary to study the competency-related factors and the change of competencies after the education program in the future. How much competency is needed depends on the clinical environment or setting, but there is no doubt that the need for informatics competencies in the healthcare environment is increasing. Therefore, exposure to nursing informatics in the course can affect the attitudes and knowledge of nursing students and informatics education provided to clinical nurses could strengthen the role of a decision maker or manager.

In addition, the information technology applied to the medical field is developing and changing rapidly. The concept and scope of nursing information competencies have been defined differently [31]. Therefore, this tool has limitations in assessing the full range of nursing informatic competencies needed in the nursing field. In future research, it will be necessary to newly consider items that can measure the core competencies of nursing informatics required in the clinical setting in accordance with the rapid change of information technology.

### Limitations

Although this study established the validity and reliability of the K-SANICS, it has some limitations. First, the generalization of our findings should be done carefully, as our data were collected from nursing undergraduates from colleges in a few regions in Korea. Second, as this was the first study to use K-SANICS, there is a lack of cumulative verification of the reliability and the validity of K-SANICS. We have identified the lack of a stability test in the form of test-retest as the third limitation of our study. Despite the limitations of this study, we believe that the K- SANICS could be useful in research as well as in the development of informatics curricula for nursing students.

## Conclusion

We expect the validated scale to contribute to improving nursing students’ and clinical nurses’ informatics competency through its use in the implementation and establishment of nursing informatics content in the clinical curriculum. Furthermore, improving the nursing informatics competency of students will contribute to them having the skills necessary to deliver good quality care as registered nurses.

## Data Availability

The datasets generated and/or analyzed during the current study are not publicly available to protect the participants but are available from the corresponding author on reasonable request.

## Abbreviations

K-SANICS: Korean- Self-Assessment of Nursing Informatics Competencies Scale
SANICS: Self-Assessment of Nursing Informatics Competencies Scale
